# Supplementary material for: Factors associated with stigma and manifestations experienced by Indian health care workers involved in COVID-19 management in India: A qualitative study
Source: Glob Ment Health (Camb). 2023 Jul 28;10:e46. doi: 10.1017/gmh.2023.40 (PMC10579676; doi:10.1017/gmh.2023.40)
Supplement: Grover et al. supplementary material [file S2054425123000407sup001.docx]

**Supplementary Table 1: Qualitative Interview Guide**

- **Impact on work life**: Question: We would like to know how your work life has changed due to COVID-19 (please elaborate) Probe issues like burden of work, stigma, work relationships, family
- **Impact on Family life**: Question: We would like to know, how your family life has changed due to COVID-19 (Please elaborate) Probe issues like time spent with family, stigma, relationships within family and so on
- **Impact sense of well-being**: Question: We would like to know, how this experience of working with COVID has influenced you personally Probe issues like sleep, eating habits, job satisfaction, overall motivation, stigma, happiness
- **Coping during COVID-19**: Question: How have you managed to cope with all the challenges you had to face due to COVID – 19 Probe issues like, sharing experiences with others (family members, friends, colleagues), exercise, music etc.
- **Suggestions on how to mitigate the stigma due to COVID-19 Question**: What interventions do they think are needed to mitigate the stigma that many health care providers are facing due to COVID-19 Probe issues like information that is needed, interventions within the health system, within families, communities, etc.
